# Macrophages and fibroblasts during inflammation and tissue repair in models of organ regeneration

**DOI:** 10.1002/reg2.77

**Published:** 2017-06-06

**Authors:** Anthony L. Mescher

**Affiliations:** ^1^ Department of Anatomy and Cell Biology, Indiana University School of Medicine – BloomingtonIndiana University Center for Developmental and Regenerative BiologyBloomingtonIN47405USA

**Keywords:** amphibian limb regeneration, fibroblast, fibrosis, inflammation, macrophage, scar

## Abstract

This review provides a concise summary of the changing phenotypes of macrophages and fibroblastic cells during the local inflammatory response, the onset of tissue repair, and the resolution of inflammation which follow injury to an organ. Both cell populations respond directly to damage and present coordinated sequences of activation states which determine the reparative outcome, ranging from true regeneration of the organ to fibrosis and variable functional deficits. Recent work with mammalian models of organ regeneration, including regeneration of full‐thickness skin, hair follicles, ear punch tissues, and digit tips, is summarized and the roles of local immune cells in these systems are discussed. New investigations of the early phase of amphibian limb and tail regeneration, including the effects of pro‐inflammatory and anti‐inflammatory agents, are then briefly discussed, focusing on the transition from the normally covert inflammatory response to the initiation of the regeneration blastema by migrating fibroblasts and the expression of genes for limb patterning.

Processes investigated by regenerative biologists differ from those in the broader field of developmental biology by invariably having tissue injury as a trigger. Any type of physical injury to multicellular animals immediately produces a cascade of enzymatic and cellular reactions within the damaged tissues known as an inflammatory response, the initial phase in processes of tissue repair and regeneration.

In injured tissues of vertebrates blood coagulation is the first overt pro‐inflammatory and wound‐healing reaction, in which extravasated platelets and plasma proteins are activated upon contact with collagen and tissue factor components of the vascular subendothelium. Such contact leads almost instantly to the proteolytic cleavage of prothrombin to thrombin, a serine‐protease which converts the fibrinogen of plasma to insoluble fibrin strands. The resulting fibrin clot quickly produces hemostasis at the injured site and serves as a depot for the activated platelets, which immediately degranulate and release various protein factors including many growth factors and chemokines that induce extravasation and chemotaxis of neutrophils, monocytes/macrophages, and other cells. In mammals platelets are small (2−4 μm), membrane‐bound cytoplasmic fragments derived from megakaryocytes in bone marrow and circulating abundantly in blood. In contrast, the blood of non‐mammals, including the fish and amphibian species widely used for studies of organ regeneration, contains no platelets, but rather larger nucleated thrombocytes. These cells usually derive from hematopoietic tissues other than bone marrow but act much like platelets after an injury, undergoing degranulation with blood coagulation and releasing bioactive factors which convert extravasated plasma into serum, setting the stage for inflammation and tissue repair (Sugimoto, 2015).

In addition to chemokines derived from platelets, nonspecific factors with damage‐associated molecular patterns (DAMPs) and pathogen‐associated molecular patterns (PAMPs), released from dying cells and microbial invaders respectively, and reactive oxygen species generated by cell injury all serve to activate both immune and mesenchymal cells locally. The patterned factors bind toll‐like receptors (TLRs) and directly stimulate recruitment, proliferation, and activation of both hematogenic and resident macrophages and other immune cells, as well as fibroblasts, mesenchymal stem/stromal cells (MSCs) and various epithelial cells which together orchestrate tissue repair, defined as restoration of tissue integrity and homeostasis (Shaw & Martin, 2016; Wynn & Vannella, 2016). DAMPs include fragments of various chromatin‐associated and cytoplasmic proteins, DNA, RNA, and purine metabolites; PAMPs, which are released from microorganisms caught up during inflammation, include fragments of nucleic acids and various cell wall components.

Active factors released locally by exocytosis from platelets and immigrating leukocytes and by dissociation from sulfated glycosaminoglycans of the fragmented extracellular matrix (ECM) promote repair of the injured tissues and defend the wounded tissues against invading microorganisms. Cells mediating an innate immune response, particularly circulating monocytes and tissue‐resident macrophages, have long been recognized as having key roles in repair (Vannella & Wynn, 2017; Wynn & Vannella, 2016) and have recently been shown to have similar functions in certain processes of organ development and regeneration (Godwin & Rosenthal, 2014; Godwin, Pinto, & Rosenthal, 2017; Mescher, Neff, & King, 2017). New work with both new and long‐established models of regeneration in mammals and amphibians is reviewed here and highlights the importance of controlling activities in macrophages and possibly other immune cells to provide a local inflammatory response adequate to initiate repair. Timely resolution of inflammation is crucial to prevent fibrotic activity and allow the possibility of inductive events leading to organ regeneration. Further discussion of immune cells in injury‐induced inflammation, along with the importance of stress for determining outcomes in repair and regeneration, has appeared elsewhere (Mescher et al., 2017).

## 1 ROLES OF MACROPHAGES AND FIBROBLASTS IN WOUND REPAIR AND REGENERATION

Mitogenic and other growth‐promoting factors released after tissue injury from activated platelets and sequestration sites in the ECM collectively produce an initial proliferative response during the early phase of inflammation which sets the stage for how the tissues will be repaired (Werner & Grose, 2003). In amputated amphibian limbs and other severely damaged organs, this wound‐associated proliferation begins primarily in fibroblastic cells of the connective tissue associated with skin, muscle, nerves, and parenchymal tissues (Gardiner, Endo, & Bryant, 2002; Wynn & Ramalingam, 2012). Subsequent activities of these fibroblastic cells vary widely along a continuum that determines the outcome of the reparative response, from fibrosis to complete anatomic and functional regeneration of the limb or other organ.

Of the many cell types active during repair of damaged tissues, macrophages have been found in many mammalian models to function as “master regulators of inflammation and fibrosis” (Wynn & Barron, 2010). Macrophages have well‐characterized dual roles during inflammation and repair as the primary phagocytes for removal of debris and apoptotic cells as part of an innate response to tissue injury and as antigen‐presenting cells which specifically activate lymphocytes for an adaptive immune response to “non‐self” antigens found locally during inflammation. Work reviewed by Wynn, Chawla, and Pollard (2013) has revealed (1) that, in their basal state, macrophages residing in different organs are not only anatomically distinct but have quite different transcriptional profiles important in maintaining metabolic homeostasis, (2) that such macrophages derive early in embryogenesis from monocytes originating in the yolk sac and have important roles in the development of the organs to which they migrate, and (3) that after injury both resident macrophages and newly arriving monocytes begin to exhibit highly flexible and shifting programs of gene expression with critical regulatory activity through all phases of repair and fibrosis.

**Figure 1:**
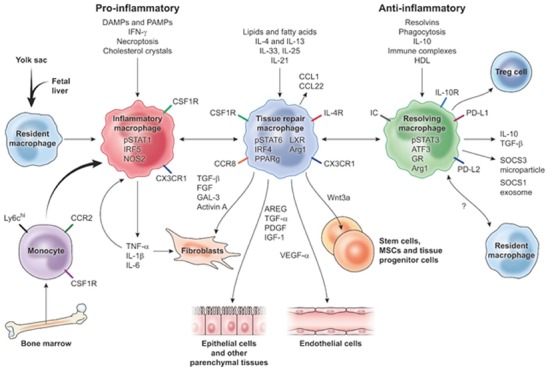
**Macrophage activation phenotypes in tissue repair, regeneration, and fibrosis** (reproduced with permission from Wynn and Vannella, 2016, *Immunity* **44**: 450–62). Throughout inflammation and tissue repair in mammals, the activation states of resident macrophages and immigrating monocytes change to promote the tasks at hand, including angiogenesis, reformation of epithelial continuity, growth and differentiation of stem cells, and stimulation of widely ranging fibroblast activities. DAMP, damage‐associated molecular pattern; PAMP, pathogen‐associated molecular pattern; T_reg_ cell, regulatory T cell; IRF5, interferon regulatory factor 5; NOS2, nitric oxide synthase 2; LXR, liver X receptor; AREG, amphiregulin; Arg1, arginase‐1; IRF4, interferon regulatory factor 4; PPARg, peroxisome proliferator‐activated receptor g; FGF, fibroblast growth factor; GAL‐3, galectin‐3; TGF, transforming growth factor; IC, immune complex; GR, glucocorticoid receptor; ATF3, activating transcription factor 3; SOCS, silencer of cytokine signaling

Knowledge of the central roles of macrophages in inflammation and regeneration is developing rapidly and numerous recent reviews are available (Chazaud, 2014; Das et al., 2015; Wynn & Vannella, 2016). Figure 1 summarizes much of the most important information on the changing macrophage phenotypes during inflammation. Immediately after tissue injury DAMPs, PAMPs, and cytokines from neutrophils trigger resident macrophages and recruited monocytes to become critical local sources of chemokines, matrix metalloproteinases (MMPs), cytokines such as interleukin‐1β (IL‐1β) and tumor necrosis factor‐α (TNF‐α), and other secreted factors that produce and coordinate the many activities of the inflammatory response. It is now clear that fibroblasts and other mesenchymal cells also respond to DAMPs and PAMPs, as well as to pro‐inflammatory factors from macrophages, triggering growth, migration, and new synthetic activities in these cells which reinforce the local immune response and other aspects of inflammation (Bernardo & Fibbe, 2013; Nowarski, Jackson, & Flavell, 2017). Prolongation of this macrophage−fibroblast activation state is characteristic of fibrosis and maladaptive repair processes (Wynn, 2008).

As inflammation proceeds normally the dominant macrophage population shifts from an “inflammatory” to a “tissue repair” phenotype characterized by secretion of various paracrine factors, such as vascular endothelial growth factor‐α (VEGF‐α), amphiregulin, transforming growth factor‐β (TGF‐β), insulin‐like growth factor‐1 (IGF‐1). Collectively these factors promote angiogenesis as well as further proliferation and fibroblastic cells, MSCs, and other local stem and progenitor cells. Fibroblasts shift from inflammation‐related, potentially fibrotic activity to the synthesis of new protein and proteoglycan ECM components for re‐establishing normal tissue structure (Karin & Clevers, 2016). After a variable period (Fig. 1), macrophages with a largely anti‐inflammatory or “resolving” phenotype, secreting TGF‐α, IL‐10, and suppressors of cytokine signaling (SOCS), become the dominant population (Das et al., 2015; Wynn & Vannella, 2016). Macrophage activity now promotes the overall resolution of inflammation and is closely coordinated with the new functions of fibroblasts and other mesenchymal cells engaged in tissue repair and differentiation.

Often described very broadly as M1 and M2 polarization, the different macrophage activation stages consist of an array of states induced by the myriad signals from the microenvironment and inflammatory stimuli, a large proportion of which converge on the PI3K/Akt pathway and its downstream targets (Vergadi, Ieronymaki, Lyroni, Vaporidi, & Tsatsanis, 2017). Dysregulation of the shifting macrophage phenotypes during repair, leading for example to excessive production of pro‐inflammatory agents or deficient generation of proresolution macrophages, can produce defective repair or regeneration, chronic wounds, or excessive fibrosis and scarring (Wynn & Ramalingam, 2012). Studies of experimental injuries in diverse organs have shown that deleting macrophages at specific times during inflammation interferes with subsequent phases and leads to defective repair (Fiore et al., 2016; Klinkert et al., 2017; Perego et al., 2016). Of particular importance to regenerative biologists is the newer appreciation of the changing phenotypes of fibroblasts and other mesenchymal cells which orchestrate the onset of regeneration (Karin & Clevers, 2016; Nowarski et al., 2017).

Recent evidence shows that during the inflammatory phase of an organ's response to injury or infection fibroblasts and other mesenchymal cells not only sense and respond to DAMPs and PAMPs via TLRs in a manner like that of innate immune cells, they are also capable of polarizing into distinct phenotypes with secretomes that enhance the immune response early in inflammation and then foster tissue homeostasis and reconstruction during inflammatory resolution (Bernardo & Fibbe, 2013). Among the mesenchymal cell subsets frequently classified as fibroblasts in adult organs are myofibroblasts and the pericytes surrounding blood capillaries, both of which prominently express α‐smooth muscle actin, as well as the multipotent MSCs, which can be expanded from the connective tissue components of skin, muscle, and fat. The range of these cells’ activation and differentiation states, together with differences in their developmental origins and specific locations within connective tissues, produce considerable heterogeneity with respect to an organ's resident mesenchymal cell population. Increasingly well studied are the heterogeneous fibroblastic cells in the connective tissue of layered organs with important barrier functions, such as the integument (Driskell & Watt, 2015) and the intestinal tract (Powell, Pinchuk, Saada, Chen, & Mifflin, 2011). Directly adjacent to the epithelium exposed constantly to potentially injurious material and microbial invasion, mesenchymal cells respond to epithelial signals and help shape the response of innate and adaptive immune cells located within that connective tissue layer (Nowarski et al., 2017).

While regulation of fibroblast proliferation, type I collagen deposition, and myofibroblast differentiation may be the most important response of mesenchymal cells to epithelial and immune signaling to avoid fibrosis and allow successful organ regeneration (Barron & Wynn, 2011), various other reciprocal interactions between macrophages and fibroblasts have been found. In the gut wall activated fibroblasts express monocyte chemotactic protein 1 for recruitment of macrophages (Kim et al., 2011) and myofibroblasts help control monocyte activation by releasing prostaglandin E2 (Roulis et al., 2014). Besides a direct role in fibrosis, pericytes in both the dermis and lungs are also important in recruiting and retaining macrophages and other inflammatory mediators (Nowarski et al., 2017). Other chemokine/cytokine pathways and small signaling factors by which mesenchymal cells affect the activities of macrophages and other immune cells have been reviewed in detail by Bernardo and Fibbe (2013). As another mechanism of this reciprocal interaction, modification of type I collagen in the ECM by a fibroblast‐derived post‐prolyl peptidase has been shown to increase macrophage adhesion and activity locally (Mazur, Holthoff, Vadall, Kelly, & Port, 2016).

Specific populations of lymphocytes, the effector cells of adaptive immunity activated by monocyte‐derived antigen‐presenting cells during inflammation, are also clearly involved in mammalian tissue fibrosis (scarring) and fibrotic disorders (Wynn, 2004). Many details of the mechanisms by which lymphocytes promote injury‐related fibrosis are active areas of investigation and beyond the scope of this review, but both T_H_2‐type and T_H_17‐type immune responses are pro‐inflammatory and profibrotic, and cytokines in the IL‐17 family have emerged as important drivers of postinflammatory fibrosis in many organs (Chizzolini & Boin, 2015; Kirkham, Kavanaugh, & Reich, 2014; Wynn & Ramalingam, 2012) and during allograft rejection (Liu, Fan, & Jiang, 2013). Cytotoxic T cells and other effectors of adaptive immunity may also directly eliminate genetically reprogrammed cells that express “embryonic” or other neoantigens recognized as “non‐self” and arise early in the transition from inflammation to regeneration (Quigley & Kristensen, 2015). Fibroblasts of the intestinal wall also express antigen‐presenting components and can directly modulate local T cell functions and activate local T cell expansion (Nowarski et al., 2017).

## 2 INFLAMMATION AND IMMUNITY IN REGENERATION WITHIN MAMMALIAN SKIN, EARS, AND DIGITS

### 2.1 Skin regeneration

Studies of “scarless wound healing” in fetal murine and ovine skin led to the concept of “skin regeneration.” The integument is typically the largest vertebrate organ and serves many diverse functions. Readily accessible and histologically complex, with its several tissue components arising via epithelial−mesenchymal interactions during development by relatively well understood inductive interactions, skin provides a useful model for analyses of organ regeneration more broadly. Fetal skin in several mammalian species was found to regenerate after incisions or full‐thickness excisions, avoiding excessive fibrosis and undergoing morphological and functional reconstitution of all epidermal components, layers, and derivatives, including hair follicles and glands (Ferguson et al., 1996; Stelnicki, Chin, Gittes, & Longaker, 1999). Studies in the 1980s also found that certain species of adult gecko lizards (genus *Geckolepis*) have the ability to autotomize the full‐thickness integument almost entirely and regenerate skin with scales rapidly without scarring, apparently from stem cells deeper in the hypodermis (Scherz, Daza, Kohler, Vences, & Glaw, 2017). Several other recent reviews have discussed various aspects of new investigations of fetal and adult vertebrate skin regeneration (Gawronska‐Kozak, Grabowska, Kopcewicz, & Kur, 2014; Leavitt et al., 2016; Takeo, Lee, & Ito, 2015).

In an early review of fetal skin regeneration, we discussed its dependence on excision rather than burn injury, its similarity to wound healing in adult mice lacking macrophages, and its dependence on local not systemic immunity (Harty, Neff, King, & Mescher, 2003). The likelihood that developmental changes in the cutaneous immune system and inflammatory response explained the increased tendency for scarring during fetal development suggested to us that the relatively inefficient adaptive immunity of urodele amphibians (newts and salamanders) may in part account for the more effective, life‐long and widespread regenerative capacity of these species compared to the phylogenically more recent anuran amphibians (frogs and toads). Fetal and adult mouse models of scarfree skin regeneration after full‐thickness wounding have not only underlined the centrality of the immune and inflammatory response in the transition to scarring, but have also identified several specific cytokines and cytokine modulators regulating synthesis of ECM components and important for scarring (Balaji et al., 2017; Gawronska‐Kozak, Bogacki, Rim, Monroe, & Manuel, 2006; Gawronska‐Kozak et al., 2014; Mirza, DiPietro, & Koh, 2009; Wilgus, Bergdall, Dipietro, & Oberyszyn, 2005; Zheng et al., 2016).

Since the review by Harty et al. (2003), scar‐free wound healing of excised skin has also been demonstrated in urodeles, along with its decline during development in both anurans and urodeles (Godwin & Rosenthal, 2014; Mescher et al., 2017; Seifert & Maden, 2014). The neotenic salamander, *Ambystoma mexicanum*, the axolotl, widely used in studies of limb regeneration, is particularly useful in studies of inflammation and skin regeneration. Both juvenile and adult axolotls show scar‐free regeneration after large, full‐thickness skin excisions (Levesque, Villiard, & Roy, 2010), but after induced metamorphosis similar wounds had significantly higher leukocyte infiltration, followed by much slower and less perfect skin regeneration (Seifert, Monaghan, Voss, & Maden, 2012b). Recently it was found that juvenile axolotls can also regenerate all components removed via punch biopsy through the soft tissues of the lower jaw, including all skin layers, muscle, and oral mucosa, with minimal overt inflammation (Charbonneau, Roy, & Tran, 2016).

An interesting mammalian model of adult skin regeneration is the African spiny mouse (*Acomys* species), which appears capable of partial skin autotomy in escaping predators. Seifert et al. (2012b) compared adult dorsal skin of two *Acomys* species and true mice (*Mus*) with regard to tensile strength, histology, and repair of small and large, full‐thickness excisional wounds. They found *Acomys* skin to have 20‐fold lower tensile strength and a significantly greater volume of variously sized hair follicles associated with large sebaceous glands. Wounds in *Acomys* were re‐epithelialized more rapidly and with much less scarring compared to similar wounds in *Mus*; contained predominately type III collagen rather than type I collagen as in *Mus*; formed very few myofibroblasts, which were abundant in wounds of *Mus*; and developed dermis and epidermis with induction and formation of new hair follicles which were absent in *Mus* (Seifert et al., 2012a).

The remarkable regenerative capacity of adult skin in this mammal was elucidated by analyzing expression profiles of inflammation‐related genes, densities of resident immune cells, and cytokine levels in *Acomys* and *Mus* dorsal skin undergoing repair. Brant, Lopez, Baker, Barbazuk, and Maden (2015) examined gene expression in the skin of the two species during the week after full‐thickness excisions. Wounding in *Mus* elicited a strong, well‐characterized inflammatory response, while that provoked in *Acomys* was substantially muted, with little or no increase in expression for most cytokines and chemokines assayed (Brant et al., 2015). Levels of *Il‐1β*, *IL‐4*, and *granulocyte colony stimulating factor 3* (*Csf3*) expression were all significantly higher in *Mus* than in *Acomys*, while those of *TGF‐β* and *cyclooxygenase‐2* (*cox2*) were higher in *Acomys*. A similar analysis of genes involved in forming and degrading ECM components showed that their expression was among the most highly upregulated in *Acomys* wounds, suggesting more active ECM turnover during *Acomys* wound healing than in *Mus*.

Immunohistological analyses by the same group provided striking evidence that, unlike skin wounds in *Mus*, macrophages were virtually absent in *Acomys* wounds, although present in the normal tissue surrounding the wound and in the spleen (Brant, Yoon, Polvadore, Barbazuk, & Maden, 2016). Levels of circulating monocytes in *Acomys* were similar to those in *Mus*, but with a higher percentage of lymphocytes and a lower percentage of neutrophils (Brant et al., 2016). As might be expected from the absence of macrophages, *Acomys* wounds were found to contain much lower levels of most common chemokines and cytokines, but not those of the IL‐1 family (Table 1). Finally, although skin wounds in both species were comparably vascularized during the first month after injury, with similar early expression of *vegfα*, *Mus* wounds produced much thicker layers of dense collagen, greater expression in most of the collagen genes analyzed, and greater cell densities in the wounds (Brant et al., 2016). The authors conclude that in *Acomys* the relative absence of macrophages in skin wounds allows wound repair without fibrosis, a situation which permits a regenerative response rather than scarring, a possibility discussed further below with the capacity of *Acomys* and immunodeficient mice for ear‐hole regeneration.

**Table 1:** **Summary of maximal cytokine levels detected in *Mus* and *Acomys* wounds 3–14 days post‐excision by mouse cytokine array** (from Brant et al., 2016, *Wound Rep. Regen*., **24**: 75–88). Levels for each cytokine are shown by differing numbers of plus signs, with five maximum, one minimum. A minus sign indicates “absent”

Cytokine	*Mus*	*Acomys*
GM‐CSF	++	−
G‐CSF	+++++	−
CCL1	+	−
slCAM‐1/CD54	++++	
IL‐2	+++	−
IL‐4	+	−
IL‐6	++++	−
IL‐7	+	−
IL‐13	+	−
CXCL10	++	−
CXCL11	+	−
CXCL1	+++++	−
M‐CSF	+++	−
MCP‐5/CCL12	++	−
MIG/CXCL9	+	−
MIP‐1β	+++++	−
TREM‐1	++++	−
TIMP	++++	−
C5/C5a	+++++	+
IL‐16	++	+
MCP‐1/CCL2	+++++	+
MIP‐1α	+++++	++++
MIP‐2	+++++	+
TNF‐α	++	+
CXCL13	+	+
IFN‐γ	+	+
IL‐1α	++	++
IL‐1β	++++	++++
IL‐1ra	++++	++++
RANTES	++	

Less dramatic than *Acomys* skin regeneration, two other related models of the hair follicle “mini‐organ” neogenesis triggered by injury have been developed in mice and both clearly require participation of local immune cells and fibroblasts. The first involves the de novo development of scattered hair follicles in the new epidermis overlying dermal scar tissue, often weeks after full‐thickness excisions of dorsal skin, a process termed wound‐induced hair neogenesis (WIHN). Like hair follicle development in the fetus, WIHN depends on the activation of canonical Wnt signaling, but recent evidence has clarified differences in the inductive events (Wang et al., 2015). WIHN begins with DAMP activation of keratinocyte TLR‐3 and its downstream effectors IL‐6 and STAT3 (Nelson et al., 2015) and is augmented by fibroblast growth factor 9 (FGF‐9) from γδ T lymphocytes in the epidermis, which activates FGF‐9 and Wnt signaling in the dermis (Gay et al., 2013). Studies of immune interactions in such a relatively simple regenerating system offer new opportunities for understanding similar activity during the initial phase of regeneration in other organs.

In another recent model, “quorum sensing regeneration” in multiple hair follicles is triggered simply by the minor injury of plucking a threshold number of hairs properly arranged among neighboring follicles (Chen, Plikus, Tang, Widelitz, & Chuong, 2016). In this system apoptotic keratinocytes in the plucked follicles release the chemokine CCL2, causing the accumulation of macrophages releasing TNF‐α which stimulates hair regeneration by activating Wnt signaling in fibroblasts (Chen et al., 2016). The interaction between monocyte‐derived cells and fibroblasts is central to this regenerative process, which may serve to model the injury effect in specific tissues of more complex regenerating systems.

### 2.2 Regeneration during ear‐hole closure

**Figure 2:**
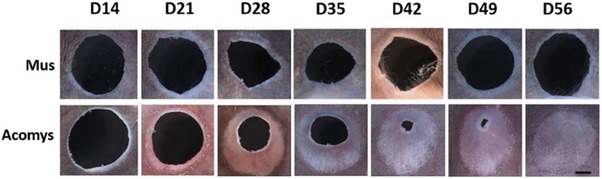
**Four‐millimeter ear punch closure in *Acomys* and *Mus* (C57BL/6) in 8 weeks post‐wounding**. 220×; scale bar is 1 mm. (Reproduced with permission from Matias Santos et al., 2016, *Regeneration* **3**: 52–61)

Regeneration of vertebrate appendages (discussed below) is often referred to as epimorphic regeneration and features the formation and growth of a blastema, a transient distal accumulation of proliferating, lineage‐restricted mesenchyme cells that reproduce the missing organ components. Large (4‐mm diameter) holes punched through adult *Acomys* ear pinnae close rapidly (Fig. 2) with regeneration of all missing tissue structures, including cartilage, dermis, hair follicles, and sebaceous glands (Matias Santos et al., 2016; Seifert et al., 2012a). Based on many criteria, including mesenchymal cell proliferation in association with innervation and wound epidermis, Seifert and colleagues concluded that regeneration of skin and other tissues in ear‐holes involves blastema growth (Gawriluk et al., 2016). They also confirmed classic studies showing similar regenerative capacity in rabbit ears and found that MRL/MpJ mice failed to regenerate 4‐mm holes, despite their well‐known capacity for epimorphic regeneration after 2‐mm ear punches (Heber‐Katz, 1999).

MRL mice and a related strain capable of 2‐mm ear‐hole regeneration, LG/J, have defects of adaptive immunity rendering them prone to autoimmune disease (Gourevitch et al., 2014; Rai & Sandell, 2014). Despite reports of enhanced regeneration in other organs, adult MRL/MpJ mice do not regenerate skin in full‐thickness, dorsal excisions (Rai & Sandell, 2014). However, adult “nude” mice, profoundly T‐cell‐deficient due to lack of the functional *Foxn* genes required for thymic development, are capable of both epimorphic regeneration during ear‐hole closure and scar‐free regeneration of dorsal skin excisions (Gawronska‐Kozak et al., 2006, 2014). Recent work implicates the absence of *Foxn1* expression in nude mouse skin with the “neotenic” state of its dermal fibroblasts and its capacity for scar‐free skin regeneration (Kur‐Piotrowska et al., 2017).

In the light of the enhanced regenerative capacity in various immunodeficient mice, it is interesting that specific defects of adaptive immunity also occur in the spiny mouse *Acomys*. In what appears to be the only analysis of *Acomys* immune activity to date, Pennello, Taylor, Matlack, Karp, and Riggs (2006) compared various adaptive immune responses in adult *Acomys cahirinus*, true mice (*Mus musculus*), immunodeficient (*xid*) mice, and gerbils (*Meriones unguiculatus*), to which *Acomys* is more closely related than to mice (Agulnik & Silver, 1996; Chevret, Denys, Jaeger, Michaux, & Catzeflis, 1993). The regeneration capacity of gerbils appears untested, but immune deficiencies rendering these rodents much more susceptible than mice or rats to various viral, bacterial, and parasitic infections make them an important model of many human diseases, e.g. Sun et al. (2016). It was found that immune cells from *Acomys*, gerbils and *xid* mice, but not from *Mus* controls, failed to respond to specific thymus‐independent type 2 (TI‐2) antigens and that *Acomys*, gerbils and *xid* mice lack a subset of B lymphocytes present in normal mice (Pennello et al., 2006). The responses of *Acomys* to TI‐2 antigens showed various similarities to those of MRL mice (Manheimer, Victor‐Kobrin, Stein, & Bona, 1984). These altered properties of lymphocytes and the near absence in *Acomys* wounds of macrophages (Brant et al., 2016) are consistent with the possibility that these immune components control activity of connective tissue cells during inflammation in a manner that allows for regeneration in *Acomys* skin and large ear‐holes. Clearly further study of immune cells and fibroblasts during the acute inflammatory response in *Acomys*, like recent work with nude mice (Kur‐Piotrowska et al., 2017), will help explain epidermal−mesenchymal interactions underlying the enhanced regenerative capacity in this mammal.

### 2.3 Digit tip regeneration

Another example of organ regeneration in mammals is the regrowth of amputated digit tips found in mice and primates (Simkin et al., 2015). In this murine model a proximal portion of the nail organ must remain after amputation for successful digit regeneration, which raises the possibility of stimulatory roles for immune cells. The amputated surface is re‐epithelized in part from the nail matrix, the proximal portion of which contains nail stem cells that generate Wnt signaling activity, an important driver of digit regeneration (Takeo et al., 2013). As discussed in more detail elsewhere (Mescher et al., 2017), there is evidence that like hair follicles the nail matrix is an immune‐privileged site. Compared to other skin on a digit, the human nail matrix and underlying connective tissue have been found to have a very low density of helper and cytotoxic T cells, natural killer (NK) cells, and mast cells; strongly downregulated expression of stimulatory components by Langerhans cells in the epithelium; and prominent local expression of a wide variety of anti‐inflammatory and immunosuppressive proteins in the proximal nail matrix (Ito et al., 2005). Cutaneous immunity has apparently not been examined in mouse digits; however, in most respects mouse nail organs are developmentally and anatomically very similar to those of primates (Fleckman, Jaeger, Silva, & Sundberg, 2013).

Ito et al. (2005) speculate that the nail immune system of humans evolved as a site of relative immune privilege suppressing local inflammatory and/or autoimmune damage because its components helped protect individuals from loss of claws (or nails) after pro‐inflammatory mechanical or environmental insults and accelerated repair and use of these appendages, limiting pain and swelling after claw trauma or infection. With the well‐established requirement for the nail organ during digit tip regeneration, the potential role of local immunity in this system seems well worthy of investigation (Saito, Ohyama, & Amagai, 2015). Studies of immune cell activities in the murine nail apparatus, their likely dependence on local Wnt signaling (Augustin et al., 2013), and their roles in modulating the acute inflammation produced by amputation are likely to provide new insights into the importance of the nail organ for digit regeneration, a clinically important model of epimorphic regeneration in mammals (Rinkevich, Maan, Walmsley, & Sen, 2015).

## 3 STUDIES OF THE INFLAMMATORY RESPONSE IN AMPHIBIAN APPENDAGE REGENERATION

Animals used to investigate limb and tail regeneration are ectothermic, with covert inflammatory responses easy to overlook, especially by developmental biologists whose expertise and interest seldom includes immunology or concepts from pathology such as inflammation. “Inflammation” does not appear in the indexes of most classic monographs on limb and organ regeneration (Carlson, 2007; Goss, 1969; Hay, 1966; Morgan, 1901; Needham, 1952; Polezhaev, 1972; Rose, 1970; Schmidt, 1968; Tsonis, 1996; Vorontsova & Liosner, 1960; Yannas, 2001). With the appearance of many inflammation‐related gene products in transcriptional and proteomic screens of regenerating tissues in fish, urodeles, and anurans, and with substantial knowledge of the changing innate and adaptive immune systems during development and metamorphosis in *Xenopus*, interest in how inflammation relates to amphibian limb and tail regeneration has grown steadily in the new century.

As the major link between innate and adaptive immune responses, macrophages are also likely to be relevant for understanding the typically diminished regenerative capacity of animals with well‐developed adaptive immunity (Mescher & Neff, 2005). Mammals, birds, reptiles, and postmetamorphic anurans, in all of which adaptive immunity is relatively rapid, efficient and multifaceted, fail completely to regenerate amputated limbs or, in the case of the most basal anurans of the genus *Xenopus*, regenerate only a tapering, skin‐covered, cartilaginous rod (Thouveny & Tassava, 1998). In contrast, experimentally amputated limbs/fins of many fish species, of larval and adult urodele amphibians, and limb buds of young, premetamorphic anurans, all of which typically lack efficient adaptive immunity, usually regenerate well, with well‐patterned reconstruction of the missing appendage. Mescher and Neff (2005) suggested that an adaptive immune response may be activated in injured tissues of nonregenerating vertebrates that leads to enhanced inflammation and fibrosis, and interferes with the developmental processes of pattern formation and organogenesis comprising regeneration. During early limb regeneration in axolotls, fibroblasts and the ECM they create provide the developmental cues needed for the localization, growth, and differentiation of cells in other lineages (Phan et al., 2015), and enhanced collagen synthesis by fibroblasts inhibits induction of a regeneration blastema (Satoh, Hirata, & Makanae, 2012). Such observations lend considerable weight to the view that even slight dysregulation of fibroblast growth and activities during the period of postamputation inflammation is likely to disrupt subsequent limb patterning.

To date the most important study on the importance of inflammatory cells for the regulation of fibroblasts in amphibian limb regeneration is that of Godwin, Pinto, and Rosenthal (2013), who broke new ground by clarifying key aspects of the urodele inflammatory response to amputation. Quantifying the accumulation of myeloid cells, the authors found increased densities of leukocytes in limb stumps within 1 day postamputation, with neutrophil density maximal by the second day and, as in mammalian wounds, macrophage density peaking a few days later. While neutrophil levels returned to that of normal limbs by day 10 postamputation, the density of macrophages remained elevated in the developing regeneration blastemas 15 days after amputation.

Using murine cytokine protein arrays, Godwin et al. (2013) detected over three dozen specific chemokines and cytokines, both pro‐inflammatory and anti‐inflammatory, of both T_H_1 and T_H_2 types, within the limb stumps, most elevated within 1 day of amputation and remaining so through at least 7 days postamputation. Induction of these chemokines and cytokines was not unique to regeneration, occurring also in limbs after crush injury or bacterial lipopolysaccharide injection. Maximal expression of major cytokines in amputated limbs was found to be generally similar to that shown in Figure 1 with the major activation phenotypes of human and murine macrophages: induction of TNF‐α, IL‐1β and IL‐6 was maximal 1–2 days postamputation, that of TGF‐β 4–6 days postamputation, and that of IL‐10 slightly later.

Specific deletion of macrophages and their monocyte precursors during the postamputation period resulted in increased levels of the inflammatory cytokines IL‐1β, TNF‐α, and interferon‐γ and reduced levels of various anti‐inflammatory and T_H_2 cytokines. Macrophage depletion also reduced expression in limb stumps of certain developmentally important genes, including *tgf‐β*, *fn* (fibronectin), *mmp‐9* and *mmp‐3*, as well as *msx2*, *prxx1*, *sp9*, *runx2*, and *dlx3*. Maximal deletion of monocytes and macrophages completely inhibited limb regeneration in all cases, amputation yielding instead fibrotic limb stumps with thick scar tissue of type I collagen (Godwin et al., 2013).

Similar leukocyte immigration and cytokine inductions were found in experiments with limbs of adult newts (Mercer et al., 2012) and larval frogs (Mescher, Neff, & King, 2013). Both of these studies also reported the local expression postamputation of other immunomodulatory factors, such as annexins, galectins, SOCS, and thymosin‐β4 (Tβ4). Similar results also occur with regenerating caudal fins of zebrafish, in which selective macrophage ablation during tissue outgrowth compromised fin patterning but did not affect the rate or extent of fin outgrowth (Petrie, Strand, Yang, Rabinowitz, & Moon, 2014). Among the functions of such macrophages in newt limb regeneration is active immunosurveillance and clearance of senescent cells generated during blastema development (Yun, Davaapil, & Brockes, 2015). Godwin and Rosenthal (2014) have speculated that one essential role of macrophages in amphibian limb regeneration may be to prevent NK‐mediated lysis of dedifferentiating progenitor cells expressing neoantigens.

In anurans the capacity for complete hindlimb regeneration declines during prometamorphosis, as manifested by regenerates with increasingly poor patterning of distal skeletal elements and fewer digits the longer amputation of larval limbs is delayed. In *Xenopus laevis* hindlimbs this regenerative loss begins at proximal levels and extends distally as the limb grows and becomes fully formed (Dent, 1962). The stages (Nieuwkoop & Faber, 1994) during which hindlimb regenerative capacity declines in *Xenopus laevis* correspond to the onset of the period in which the immune system is extensively remodeled and adaptive immune components become much more like those of mammals (Robert & Ohta, 2009). For these reasons limbs of larval anurans can be particularly useful in testing the hypothesis that activities of adaptive immune cells interfere with normal patterning and thus complete regeneration of complex organs.

The epidermis of postmetamorphic anuran skin contains a reticulum of dendritic cells resembling Langerhans cells, the monocyte‐derived resident phagocytes and antigen‐presenting cells of mammalian epidermis (Carrillo‐Farga, Castell, Perez, & Rondan, 1990), which in developing hindlimbs appear to differentiate gradually, like other limb cells, in a proximal‐to‐distal direction (Mescher et al., 2007). Future work with larval anuran limbs should take into account these resident immune cells and study their activation together with the functional states of the differentiating dermal fibroblasts during limb development and after amputation, in the transition from inflammation to regeneration or fibrosis. Preliminary work has demonstrated inflammatory cells in the distal wound epithelium of amputated larval anuran hindlimbs at both regenerating and nonregenerating stages (Alibardi, 2016; Mescher et al., 2007) and their effects on epithelial signaling is worthy of investigation.

Previous studies with larval *Xenopus* limbs support the hypothesis that metamorphic changes in the immune system, local or systemic, change the inflammatory response of limb tissues to amputation in ways that preclude limb regeneration. At developmental stage 55 or 57, in which regenerative capacity is reduced, expression of factors that promote resolution of inflammation, including annexin‐1, fibrinogen‐like protein 2, and SOCS1 and 3, is greater and more persistent compared to their expression in regeneration‐competent stage 53 (Mescher et al., 2013). We also found low levels of expression of these factors in unamputated limbs at stage 55 and/or 57, but not at stage 53, supporting the immunohistochemical evidence that immune cells become resident in differentiating limb tissues where they are available to affect the tissue response to amputation differently at different larval stages.

In regenerating stage 53 hindlimbs, downregulation of inflammatory and proresolution factors 1−4 days after amputation accompanies upregulation of several genes required for limb tissue patterning (*msx1*, *shh*, *sall1*, *hoxa13*) (Mescher et al., 2013). Persistent postamputation expression of inflammatory and proresolution factors at later, regeneration‐incompetent stages suggested prolonged inflammation and was accompanied by inhibited or attenuated expression of the pattern‐related genes. Together these data suggest that local inflammatory activities triggered by amputation must be resolved before at least some of the cell−cell signaling and inductive events required for blastema formation and patterning can occur.

**Figure 3:**
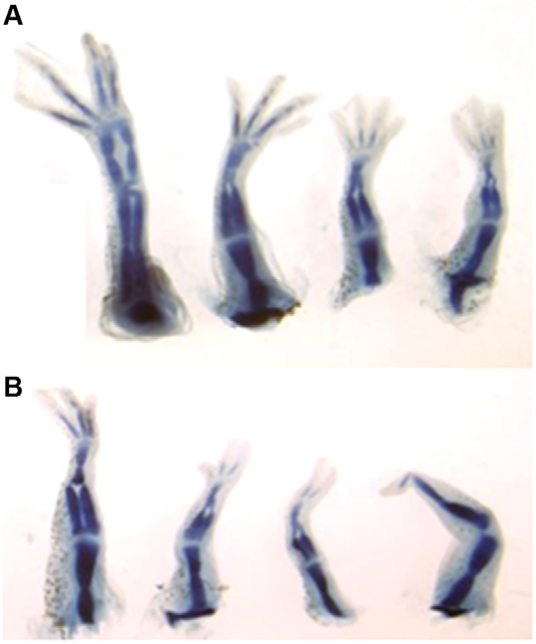
**Effects of celecoxib on regeneration of developmental stage 54/55 *Xenopus* larvae hindlimbs**. Treatment of larvae with 1 μM celecoxib, a cyclooxygenase‐2 (COX‐2) inhibitor, for 7 days post‐amputation allowed better patterning of regenerated limbs (A) than that of controls (B). (See King et al., 2012, *Anat. Rec*. **295**: 1552−61, for similar quantitative data with this and other immunomodulating agents.) Importantly, improved patterning with celecoxib or other agents was *not* found with larvae at later developmental stages, consistent with the view that additional monocyte/macrophage growth and development beyond stage 54/55 may lead to a profibrotic inflammatory environment post‐amputation not reversible by COX‐2 inhibition

Treatment of skin wounds in mice with anti‐inflammatory agents can reduce scarring and lead to more complete skin regeneration (Wilgus, Vodovotz, Vittadini, Clubbs, & Oberyszyn, 2003). With larval *Xenopus* we found that regeneration of distal limb structures was improved in stage 54/55 hindlimbs (but not in later stages) by treatment with the COX‐2 inhibitor celecoxib (Fig. 3) (King, Neff, & Mescher, 2012). Similar results were obtained with diclofenac, another COX‐2 inhibitor, and with celastrol, an inhibitor of nuclear factor‐κB (NF‐κB) activation. Although the molecular basis of these effects is not clear, they suggest that immune regulation and induced immunotolerance will probably play key parts in future attempts to promote regeneration in vertebrates that have lost this capacity.

An excellent example of this are the gain‐of‐function experiments showing the ability of postmetamorphic *Xenopus* limbs to regenerate digit‐like structures after receiving allografts of larval limb progenitor cells with constitutively active Wnt signaling and added morphogens (FGF‐10 and Shh) (Lin, Chen, & Slack, 2013). As discussed elsewhere (Mescher et al., 2017), multidigit regeneration in this work occurred *only* if the hosts were thymectomized before metamorphosis, rendering the recipient limbs largely devoid of lymphocytes. Most importantly, additional treatment of the grafted cells with the immunomodulatory peptide Tβ4 reduced apoptosis, doubled the proliferation rate of the grafted mesenchymal cells, and improved bone formation and limb patterning (Lin et al., 2013). The authors note that Tβ4 is an anti‐inflammatory agent but new evidence shows that the peptide's proresolution effects also promote regeneration in the hearts of both zebrafish and mice (Evans et al., 2013).

If regeneration can be improved by dampening inflammation, does persistent inflammation interfere with developmental events in regeneration? Single applications to freshly amputated, regeneration‐competent larval *Xenopus* hindlimbs of solutions containing pro‐inflammatory agents such as lipopolysaccharide, Freund's adjuvant, or nickel ions were found to increase *IL‐1β* expression temporarily, but had no effect on such limbs’ regenerative capacity (unpublished). A single very brief, localized application of beryllium (Be) ions in solution to newly amputated urodele limbs has long been known to inhibit limb regeneration and generate fibrosis locally (Scheuing & Singer, 1957; Thornton, 1951). The deleterious biomedical effects of Be are now recognized as primarily due to chronic inflammation produced locally by the metal's immunoadjuvant properties and great persistence in exposed tissues (Cummings, Stefaniak, Virji, & Kreiss, 2009; Sawyer, Abraham, Daniloff, & Newman, 2005a). Brief application of Be to regeneration‐competent *Xenopus* limb stumps caused expression of *IL‐1β* (normally completed within 1 day postamputation) to persist and increase for at least a week and completely inhibited regeneration in a dose‐dependent manner (Mescher et al., 2017). Expression of the immunosuppressive factor *fgl2*, which diminished by day 5 of regeneration in controls, also remained maximal on day 7 following Be treatment and similar expression patterns were found for *mmp9* and *C3* (complement component 3), further suggesting a persistent inflammatory state. All of these genes were expressed largely in immigrating leukocytes (Mescher et al., 2013), except for *C3*, which was also found expressed in blastema and wound epithelial cells (Del Rio‐Tsonis, Tsonis, Zarkadis, Tsagas, & Lambris, 1998; Mescher et al., 2013).

Expression of genes involved in limb patterning was strongly inhibited by Be, while expression of the genetic reprogramming marker *sall4* was largely unaffected (Mescher et al., 2013). This suggests that, despite unresolved inflammation, cellular dedifferentiation and reprogramming still occur locally, but that the tissue interactions and signaling required for limb patterning cannot be established, leading to regenerative failure. The acute response required to initiate repair or regeneration must at least begin to be resolved if the developmental mechanisms underlying regenerative growth are to be established. Given the key importance of fibroblasts derived from dermis and other limb connective tissues in patterning the regeneration blastema (Bryant, Endo, & Gardiner, 2002), together with control of fibroblast synthetic activity and proliferation by macrophage cytokines (Karin & Clevers, 2016; Wynn & Ramalingam, 2012), fibroblastic cells are likely to be key targets during the local response to amputation, with dysregulated inflammation tending toward fibrosis rather than blastema patterning.

It is of considerable interest that larval limbs of *Xenopus* are much more sensitive to the effects of Be than similarly sized limbs of axolotls. Brief contact of amputation wounds with 10 mM BeSO_4_ caused visible local inflammation in premetamorphic *Xenopus* limbs and inhibited regeneration, but similar treatment of larval axolotl (3−4 cm) limb stumps had no visible effects and all regenerated normally. With the urodele limbs only a 10‐fold higher dose of Be consistently inhibited regeneration. However, brief application of 100 mM BeSO_4_ to the amputation surface of anuran larvae invariably produced rapid systemic inflammation and death, while no axolotls died after Be exposure at any concentration (Mescher et al., 2013). It is noteworthy that in the early experiments with Be salts and regeneration in *Ambystoma*, larvae were completely immersed in the solutions for short periods (Thornton, 1951; Tsonis, English, & Mescher, 1991); similar immersion of larval *Xenopus* was rapidly lethal, with widespread edema and inflammation. As discussed previously (Mescher et al., 2013, 2017), urodeles’ greater tolerance of Be, like their longer tolerance of allografts compared to adult frogs or mammals, probably reflects their relatively weaker cellular immunity (Barlow, DiMarzo, & Cohen, 1981; Cohen, 1971; Tournefier et al., 1998), which includes their low MHC II diversity and weak T helper cells (Sammut et al., 1999).

Cook and Seifert (2016) further examined the inhibitory effects of 100 mM BeN on *Ambystoma* limb regeneration, immersing larger (7−8 cm) animals for 2 min immediately after amputation. Unlike our results with the much smaller larvae (Mescher et al., 2013; Tsonis et al., 1991), limbs in the larger axolotls did regenerate after Be exposure but with severely perturbed skeletal patterns. Leukocyte infiltration of the regenerating limbs was not stimulated by Be which, however, was found to inhibit migration and proliferation of both dermal fibroblasts and blastema cells, with no increase in apoptosis and without affecting either migration or growth of epidermal cells (Cook & Seifert, 2016). The results were interpreted to suggest that in urodeles Be disrupts regenerative patterning of limbs by directly interfering with mesenchymal cell or fibroblast migration and growth, a conclusion consistent with the effects of Be infused into regenerating limbs of adult newts (Scheuing & Singer, 1957).

Tail regeneration in the *Xenopus* tadpole has provided another interesting model for study of the immune system's importance for regeneration. As in most anuran species, it occurs after tail amputation at any prometamorphic stage, except for a refractory period during developmental stages 45–47 (Beck, Christen, & Slack, 2003). The response of the tail to resection before and during this period has been extensively studied by Takeo Kubo and colleagues, whose work suggests a role for immunotolerance mediated by regulatory T cells (T_reg_s) for tail regeneration in this species. During the regeneration‐refractory period expression of T cell markers in the tadpoles increases rapidly, with changes in the local, injury‐induced expression of several immune‐related genes following tail amputation (Fukazawa, Naora, Kunieda, & Kubo, 2009). Notably, expression of the specific T_reg_ marker *foxp3* was strongly upregulated within hours of amputation at regeneration‐competent stage 52/53, but not during the preceding refractory stages. (In larval *Xenopus* hindlimbs, expression of *foxp3* was upregulated transiently within a few hours of amputation and persistently in Be‐treated limb stumps at both regeneration‐competent and ‐incompetent stages, and like the other markers of immunomodulation was also prominent in unamputated limbs at stage 57 [Mescher et al., 2013).

Fukazawa et al. (2009) suggested that tail regenerative ability is restored as T_reg_s become available to suppress activities of effector lymphocytes inhibitory to regeneration during the refractory period. Consistent with this view, tail regenerative ability was significantly greater during the regeneration‐refractory stages in PU.1‐morphant tadpoles, which effectively lacked lymphocytes and other leukocytes. Moreover, tail regeneration during the refractory period of normal larvae was improved by treatment with various immunosuppressants, including celastrol, cyclosporine A, IKK inhibitor VII, or FK506 (Fukazawa et al., 2009), consistent with the view that autoreactive immune cells developing during the refractory period recognize tail blastema cells as “non‐self” and inhibit regeneration at this time, with the development of T_reg_s and the establishment of a local immune regulatory system permitting regeneration again at later stages. Local immune regulation by T_reg_s has been found to promote regeneration in several mammalian tissues (Castiglioni et al., 2015; Gandolfo et al., 2009; Liu et al., 2013; Lu et al., 2013; Zhao et al., 2012). In most of these studies the anti‐inflammatory or immunosuppressive effects of T_reg_s were either directly implicated or assumed to be part of their proregenerative activity, but other groups have reported unique populations of T_reg_s that stimulate repair directly, through the production of the epidermal growth factor family member amphiregulin (Arpaia et al., 2015; Burzyn et al., 2013).

Work on the tadpole tail regeneration model showed further that the immune suppressant FK506 rescues regenerative capacity during the refractory period if given for just the first 12 h postamputation, and that this period is characterized by expression of a gene similar to *phyH* which is downregulated by FK506 (Naora, Hishida, Fukazawa, Kunieda, & Kubo, 2013). *phyH* encodes an enzyme (phytanoyl‐CoA hydroxylase) that associates with an FK506 binding protein, and may be a useful marker for autoimmune cells inhibiting *Xenopus* tail regeneration. Immune‐related genes expressed by proliferating *Xenopus* tail blastema cells include *IL‐11* and “*cd200like‐related*” (Tsujioka, Kunieda, Katou, Shirahige, & Kubo, 2015). CD200, an immunoglobulin‐type cell surface protein, is a well‐characterized tolerance‐signaling molecule which in murine and human trophoblast and decidua promotes local generation of T_reg_ subsets, helping by a variety of mechanisms to prevent spontaneous abortions (Clark, Arredondo, & Dhesy‐Thind, 2015). On various mammalian cells, CD200 interacts with its receptor on T_reg_s and other myeloid cells to downregulate immune cell functions in many inflammatory diseases (Vaine & Soberman, 2014). Recent experiments with the tail regeneration model also implicate local expression in several tissues of long pentraxins, a well‐known group of IL‐1‐inducible proteins which regulate activities of antigen‐presenting cells and exert other diverse functions during inflammation (Hatta‐Kobayashi et al., 2016).

It should be noted that regeneration studies involving *Xenopus* hindlimbs and tails at prometamorphic larval stages may be affected by the fact that immunological development appears to proceed independently of growth and external morphological changes at these stages (Ruben, 1970; Ruben, Stevens, & Kidder, 1972). The observation that sibling tadpoles attain different developmental stages before and during metamorphosis despite being the same chronological age is well known. However, Ruben showed that activity of cells mediating the immune response to tissue allografts in such larvae was always equivalent to that of the most morphologically advanced members of the population (Ruben et al., 1972). Similar results with differentiation of lymphocytes in age‐matched larvae have also been reported during the period when hindlimb regeneration declines (stages 54–58), even following experimental delay of metamorphosis (Rollins‐Smith, Flajnik, Blair, Davis, & Green, 1997). For example, adaptive immunity in a stage 50 larva resembles that of its most morphologically developed sibs, which may be at stage 55. Variable results in larval *Xenopus* hindlimb regeneration studies among animals in sibling populations and between different laboratories often complicate interpretation of such experiments (Nye & Cameron, 2005; Slack, Beck, Gargioli, & Christen, 2004). The continued growth, development, and maturation of lymphocytes while external development is delayed by suboptimal nutrition, overcrowding, and other types of stress in laboratory conditions may help explain the slow and hypomorphic regenerative response seen in some larvae compared to age‐matched controls. As Ruben et al. (1972) point out, larvae showing rapid developmental progress should be selected for studies of regeneration or other aspects of normal *Xenopus* development.

The laboratory of Elly Tanaka has recently undertaken searches in axolotl appendages for “injury signals” or regeneration‐initiating molecules capable of triggering sustained cell activities that lead to blastema growth and patterning (Tanaka, 2016), a quest that might be expected to uncover factors released during inflammation. Using an expression cloning strategy followed by in vivo gain‐ and loss‐of‐function assays, this group identified a MARCKS‐like protein (MLP) as a potent, extracellularly released mitogen for myotubes and muscle‐derived cells (Sugiura, Wang, Barsacchi, Simon, & Tanaka, 2016). *AxMlp* expression is maximal 12–24 h after amputation in both limbs and tails, with juxtamembranous localization in cells of the newly formed wound epidermis (suggesting phosphorylation and release by those cells), and is required for normal tail regeneration (Sugiura et al., 2016). Studying movements of dermal fibroblasts and other connective tissue progenitor cells during blastema formation in an amputated axolotl digit, others in the Tanaka laboratory found that platelet‐derived growth factor BB (PDGF‐BB), but not other chemotactic growth factors such as FGF‐10, stroma‐derived factor‐1, and bone morphogenetic protein 2/4, induced migration of these cells required to establish the early blastema (Currie et al., 2016).

Released during blood coagulation and expressed subsequently by macrophages with the repair‐promoting phenotype (Fig. 1), PDGF is important in all healing wounds. The report of Currie et al. (2016) suggests that sustained production of PDGF to enhance fibroblast migration is a required activity of the local macrophages critical for blastema formation and distal outgrowth instead of scarring. This possibility is supported by the observations that fibroblast migration in amputated limbs of axolotls is severely reduced both by treatment with either a PDGFR inhibitor (Currie et al., 2016) or Be (Cook & Seifert, 2016), since exposure of macrophages to Be induces a persistent inflammatory phenotype, upregulating production of TNF‐α and reactive oxygen species, and is proapoptotic for these cells (Sawyer et al., 2005b). Both of these new regeneration studies with axolotl limbs support the view that PDGF production by macrophages is one of their functions required for the fibroblastic movements which orchestrate growth and development of other lineage‐specific blastema cells, a function at least partially inhibited by persistent inflammation in the presence of Be, interfering with successful regeneration.

## 4 CONCLUSION

As in models of organ regeneration with direct clinical relevance, replacement of amputated appendages in fish and amphibians is now clearly recognized to depend on both the immune and mesenchymal cells present in the injured tissues during the transition from inflammation to regeneration. As with many mammalian models of organ regeneration, the shifting phenotypes and regulatory activities of local macrophages and fibroblasts are likely to play several roles in resolving the initial inflammatory processes and setting conditions necessary for epimorphic regeneration. The increasingly well recognized importance of fibroblasts and their ECM products for establishing the regeneration blastema highlights the requirement that proliferation and gene activity in these cells be tightly regulated, at least in part by factors derived from macrophages, to prevent fibrosis and provide a suitable framework for the growth and development of other cell lineages during regeneration. As major antigen‐presenting cells, macrophages also control activation of lymphocytes, the diverse cells that mediate adaptive immunity. Many activities of lymphocytes are undeveloped or defective in animals with exceptional regenerative capacity and there is evidence that this may also hold true for *Acomys*, the gerbil‐like rodent with a remarkable property of skin regeneration. Further investigations of inflammation and interactions between immune and mesenchymal cells in animals with good organ regeneration ability will provide more complete understanding of the phylogenic basis of the regeneration process and yield important insights leading to improved functional restoration after clinically relevant organ damage.
